# Pelvic floor muscle training alone or in combination with oxybutynin in treatment of nonmonosymptomatic enuresis. A randomized controlled trial with 2-year follow up

**DOI:** 10.31744/einstein_journal/2019AO4602

**Published:** 2019-06-17

**Authors:** Renata Martins Campos, Adélia Correia Lúcio, Maria Helena Baena de Moraes Lopes, Claudia Rosenblatt Hacad, Maria Carolina Ramos Perissinotto, Howard I. Glazer, Carlos Arturo Levi D’Ancona

**Affiliations:** 1 Universidade Estadual de Campinas, Campinas, SP, Brasil.; 2 Hospital Universitário Maria Aparecida Pedrossian, Universidade Federal de Mato Grosso do Sul, Campo Grande, MS, Brasil.; 3 Universidade de São Paulo, São paulo, SP, Brasil.

**Keywords:** Pelvic floor/physiopathology, Nocturnal enuresis, Oxybutynin, Urinary incontinence, Child, Conservative treatment, Diafragma da pelve/fisiopatologia, Enurese noturna, Oxibutinina, Incontinência urinária, Criança, Tratamento conservador

## Abstract

**Objective:**

To compare the results of the standard urotherapy alone and associated with pelvic floor muscle training alone, and in combination with oxybutynin in treatment of nonmonosymptomatic nocturnal enuresis.

**Methods:**

A total of 38 children aged 5 to 10 years were randomized into three groups: Group I (n=12) that was submitted to standard urotherapy; Group II (n=15), standard urotherapy associated with pelvic floor muscle training; and Group III (n=11), standard urotherapy associated with pelvic floor muscle training and oxybutynin; the treatment lasted 12 weeks. The assessment tools used were playful bladder diary, and a 48-hour bladder diary, before and after treatment. After 2 years, patients were assessed by telephone using a standardized questionnaire.

**Results:**

The data of children from the three groups were homogeneous at baseline. After 12-week treatment, all children showed improved symptoms and signs of nonmonosymptomatic nocturnal enuresis, but the differences were not significant among the groups. After 2 years, the three groups showed maintenance of treatment results, but no differences among them.

**Conclusion:**

All treatment modalities were effective regarding improved enuresis and lower urinary tract symptoms, but the sample was not large enough to show differences among groups.

## Resumo

**Objetivo:**

Comparar os resultados da uroterapia padrão isolada e associada ao treinamento dos músculos do assoalho pélvico isoladamente e em combinação com a oxibutinina no tratamento da enurese noturna não monossintomática.

**Métodos:**

Trinta e oito crianças entre 5 e 10 anos de idade foram randomizadas em três grupos: Grupo I (n=12) realizou uroterapia padrão; Grupo II (n=15) realizou uroterapia padrão associada ao treinamento muscular do assoalho pélvico; e Grupo III (n=11) realizou uroterapia padrão associada ao treinamento muscular do assoalho pélvico e oxibutinina. O tratamento teve duração de 12 semanas. Os instrumentos de avaliação foram diário miccional lúdico e diário miccional de 48 horas, antes e depois do tratamento. Após 2 anos, os pacientes foram avaliados por telefone, usando um questionário padronizado.

**Resultados:**

Os dados das crianças dos três grupos eram homogêneos no início do estudo. Após 12 semanas de tratamento, todas as crianças apresentaram melhora em relação aos sinais e sintomas de enurese noturna não monossintomática, mas as diferenças não foram significativas entre os grupos. Depois de 2 anos, os resultados do tratamento se mantiveram nos três grupos, mas não houve diferenças entre os grupos.

**Conclusão:**

As três modalidades de tratamento foram eficazes na melhora da enurese e dos sintomas do trato urinário inferior, mas o tamanho da amostra não foi grande o suficiente para mostrar diferenças entre os grupos.

## Introduction

Enuresis is a common condition and its prevalence varies from 5 to 10% in children aged 6 to 7 years. Approximately 0.5 to 1% of adults maintain enuresis symptoms. Its cause is defined as immaturity of the nervous systems.^(1)^ It is defined by the International Children’s Continence Society (ICCS) as a symptom, and a condition of intermittent incontinence that occurs during sleep. It is divided into two subgroups, according to occurrence − concomitant or not − of other lower urinary tract symptoms (LUTS). When the child presents enuresis associated with LUTS, such as increased voiding frequency, incontinence, urgency, nocturia, hesitancy, straining, weak stream, intermittency and dysuria, is defined as non-monosymptomatic nocturnal enuresis (NMNE); when enuresis is not associated with LUTS it is defined as monosymptomatic.^(2)^

A guideline published by the ICCS in 2013 recommended how to evaluate and treat children with NMNE. Conservative treatments are recommended as first line, although authors warn that this guideline is not a systematic review, but it is based on good practices due to the lack of scientific evidence in this filed.^(3)^

Urotherapy is defined by ICCS, and divided into two modalities: standard therapy, including information about lower urinary tract function, behavioral modification, lifestyle advice, registration of symptoms and voiding habits, and regular support by caregivers. The other modality of urotherapy consists of specific interventions, including pelvic floor muscle training (PFMT).^(2)^

Only three studies were found in the literature on conservative treatment of NMNE; two are retrospective and the third is a randomized controlled trial. Ferández-Pineda et al., published^(4)^ their biofeedback and electrostimulation results in the treatment of NMNE and demonstrated an average of 80% of improvement of these patients. A more recent retrospective study investigated the effects of 6-month PFMT in treatment of NMNE, and found a significant improvement in the associated symptoms of enuresis, urinary incontinence, dysuria, urgency, urge incontinence, and holding maneuvers.^(5)^

Antimuscarinic drugs are employed in the treatment of LUTS with encouraging results,^(3)^ as reported by Campos et al.,^(6)^ in a study comparing PFMT with the use of oxybutynin in children with NMNE. The PFMT group showed better results in the reduction of incontinence episodes.

Although the studies to date have shown encouraging results with conservative treatments, several therapeutic modalities were used at the same time, and no individual conclusion can be drawn about how much each modality can contribute to the management of NMNE.

## Objective

To analyze the results of behavioral therapy, urotherapy, and urotherapy combined with oxybutynin; compare the efficacy of three treatment modalities; and verify the influence of time in each modality, in nonmonosymptomatic enuresis patients.

## Methods

A randomized controlled trial was conducted at the urology clinic of the *Hospital de Clínicas* of *Faculdade de Ciências Médicas* of *Universidade Estadual de Campinas* (Unicamp). This clinical trial was registered under the number CAAE: 0377.0.146.000-08, approved by the Research Ethics Committee of the university (protocol number: 465/2008), and written consent of the parents or legal guardians of the child was obtained before beginning the study.

The inclusion criteria were children with nocturnal enuresis and LUTS (assessed by a 48-hour bladder diary), aged 5 to 10 years, no previous bladder surgery, availability to attend treatment sessions, and no urinary tract infection, confirmed by laboratory tests. The exclusion criteria comprised unsigned informed consent, presence of neurogenic bladder, and prior urotherapy.

By means of a software, the participants were randomized into three groups: Group I (n=12) that was submitted to standard urotherapy (Control Group); Group II (n = 15), standard urotherapy associated with PFMT; and Group III (n=11), standard urotherapy associated with PFMT and oxybutynin.

**Figure 1:**
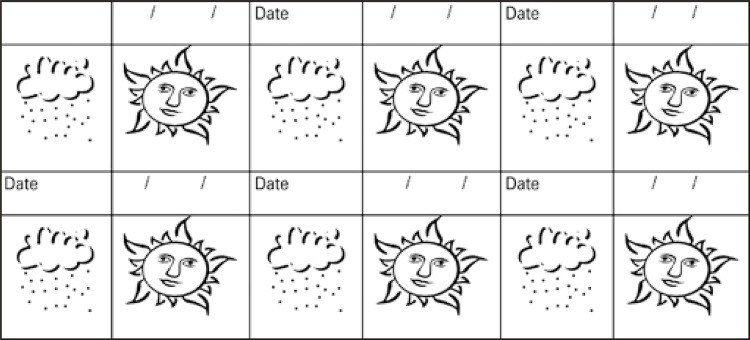
Playful bladder diary

A playful diary was used as primary tool to evaluate the effect of treatment in NMNE. The data were collected 7 days before treatment and later, monthly, until completing 12 weeks of treatment. The playful bladder diary consisted of coloring every day a sun for dry nights, or a cloud for wet nights (Figure 1). The child was instructed to color the figures alone, but the assistance of parents was allowed in case of doubts.^(6)^

A 48-hour bladder diary was used as an inclusion criterion to screen patients with LUTS, and as secondary assessment tool. Two years after the end of treatment, the participants were assessed by telephone about LUTS, using a specific questionnaire. During these 48 hours, patients were asked to take notes of the amount and type of liquid intake, presence or not of dysuria, nocturia, urgency, voiding frequency >10 times, holding maneuvers, constipation and presence of encopresis.

The bladder diaries were assessed by a physical therapist, who was blinded as to participant’s group allocation.

**Figure 2:**
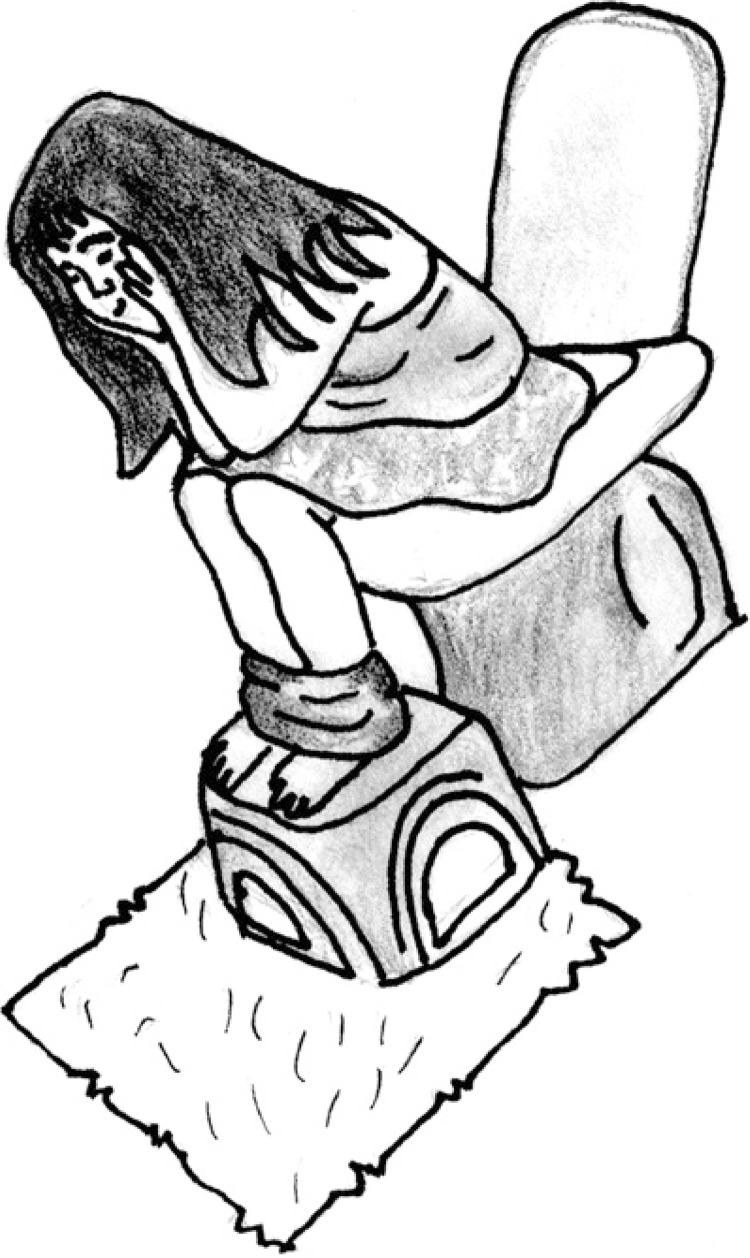
Proper voiding posture for girls

All volunteers, of both sexes, followed standard urotherapy:

- Behavioral modification: according to volume intake, measured by the 48-hour bladder diary, the amount and types of liquid the child drank during the day were obtained. The volume intake was adjusted by dividing the total volume of liquids into colored bottles of 500mL, and the participants should drink 50% of this volume during the morning, 40% in the afternoon, and 10% at night, avoiding any consumption one hour before going to bed. The children were encouraged to avoid soft drinks at night and, if possible, drink them only on weekends. Juices were allowed during the day, and caffeinated drinks were allowed only in the morning.

- Proper voiding posture: the toilet posture was explained using seat reducers and a footrest, which allowed comfortable hip abduction, trunk tilted forward, elbows supported on the thighs and underwear pulled down the ankles (Figure 2).

- Information: the child and caregivers were given information about pelvic floor muscle anatomy and function, as well as on their relation with the lower urinary tract.

- Voiding intervals were recommended at every 2 hours, starting from the first micturition episode of the day. Using a watch or a cell phone the child was instructed to register the micturition intervals alone, but parents’ help was allowed in case of any doubts.

- Bowel habits: the importance of consuming fibers for good bowel functioning was explained to children and caregivers.

Besides the standard urotherapy, Groups II and III performed PFMT and were instructed to perform a proper pelvic floor muscle contraction, without contracting the hip muscles or gluteus. In a supine position, they performed 2 series of 10 maximal effort pelvic floor muscle contractions, totaling up 20 contractions per session. Electromyography biofeedback was used to assist participants and provide motivation. Electromyographic signals from pelvic floor muscles were obtained using surface electrodes placed at the 3 and 9 o’clock positions, around the anus (over the external anal sphincter muscle) and were coupled to FlexComp Infiniti System with Biograph Infiniti Software − Thought Technology, Ltd. (Montreal, Canada). All participants were instructed to repeat the same series of ten maximal effort pelvic floor muscle contractions, as learned during the intervention, three times a day, at home.

For group III, the dose of oxybutynin used was 0.2mg/kg, twice a day.

The intervention lasted 12 weeks, and the participants attended one session per week.

Two years after the end of the treatment, children’s parents were contacted by telephone, and the following questions were asked:

1. Does your child leak, or wet, their underwear during the day? Information about the presence, or not, of incontinence.
2. Does your child leak while sleeping? Information about the presence, or not, of enuresis.
3. Do you notice the presence of stool in the child’s underwear? Information about the presence, or not, of encopresis.
4. Does your child defecate every day? Information about the presence, or not, of constipation.
5. Does your child have a sudden desire to void? Information about the presence, or not, of urgency.
6. How many times your child urinate during the day? Information about the presence, or not, of urinary frequency.
7. Does your child cross legs or squat when fell desire to pass urine? Information about the presence, or not, of holding maneuvers.

The statistical analyses were performed using SAS System Windows (Statistical Analysis System) software. A pilot study was carried out for power calculation, and the three groups were compared according to number of colored suns at the playful bladder diary. Five volunteers were enrolled in Group I; 9 children in Group II; and 21 in Group III. A SAS software called fpower was used to define sample size, establishing alpha at 5% and power at 90%. Sixteen patients were required for each group.

Due to the lack of normal distribution of the variables, non-parametric tests were used. The significance level was set at p<0.05. Mann-Whitney test was used to compare baseline measures. To compare outcomes before and after the intervention in each group, a Kruskal-Wallis test was used. To compare treatment outcomes among the three groups, variables were transformed into ranks and repeated-measures analysis of variance (ANOVA) was used. Fisher’s test was employed to compare proportions.

After treatment, the number of participants presenting dry nights and symptoms was transformed into percentage, so as to follow the ICCS recommendation of treatment outcome. According to this recommendation, it is considered no response to treatment when the participants present less than 50% reduction in symptoms; partial response considered the reduction of symptoms by 50% to 99%; and complete success occurs when 100% of symptoms are relieved. Furthermore, in the follow-up assessment, it was considered “complete success” only for those who had no relapse for 2 years after the end of treatment.^(2)^

## Results

Out of 62 eligible children 38 participated in this study. Fourteen patients were excluded because they could not attend sessions once a week, eight children refused to participate, and two caregivers refused to fill in the bladder diaries. Thus, the 38 remaining patients included in this study were divided into three groups: Group I had 12 subjects (8 girls and 4 boys; median age 9.5 years); Group II, 15 (7 girls and 8 boys; median age 7); Group III, 11 (6 girls and 5 boys; median age 8).

**Figure 3:**
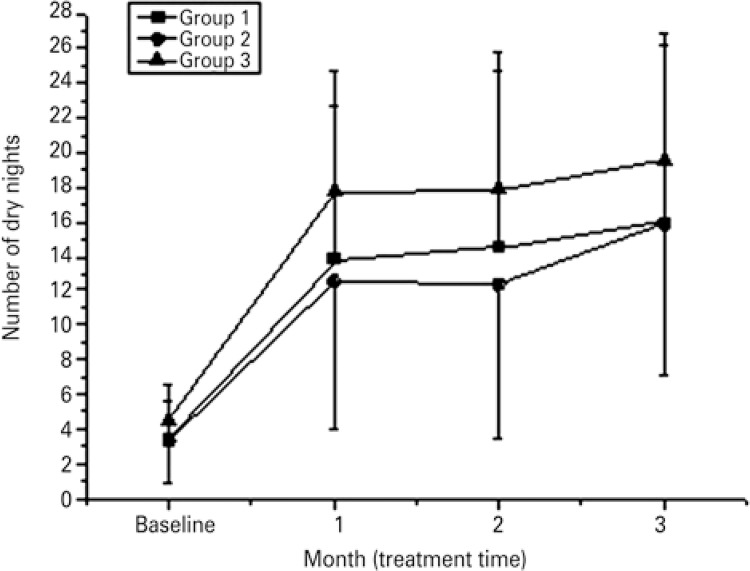
Median of the number of dry nights measured by the playful bladder diary, assessed 7 days before the beginning of treatment (baseline), after 4 weeks of treatment (Time 1), after 8 weeks of treatment (Time 2) and after 12 weeks of treatment (Time 3)

No differences were found in relation to age, sex and baseline symptoms, assessed by the two bladder diaries among the three groups. The results of the playful bladder diary are showed in figure 3, and the results of 48 hours bladder diary is showed in table 1.

**Table 1:** Median values of number and percentage of children in each group presenting symptoms before treatment and at 2-year follow-up

Symptoms	Group I		Group II		Group III		p value
		
	Baseline	2 years	Baseline	2 years	Baseline	2 years	
Dysuria	10 (83)*	1 (13)*	10 (67)*	1 (8)*	6 (55)*	1 (14)*	0.34^†^
Nocturia	9 (75)*	1 (13)*	8 (53)*	0 (0)*	8 (73)*	1 (14)*	0.72^†^
Urgency	10 (83)*	2 (25)*	14 (93)*	5 (38)*	9 (82)*	2 (29)*	0.59^†^
Voiding frequency >10	7 (58)*	3 (38)*	9 (60)*	5 (38)*	9 (82)*	2 (29)*	0.83^†^
Holding maneuvers	9 (75)*	0 (0)*	14 (93)*	1 (8)*	9 (82)*	0 (0)*	0.47^†^
Constipation	6 (50)*	1 (12)*	9 (60)*	0 (0)*	5 (45)*	1 (14)*	0.75^†^
Encopresis	8 (67)*	4 (33)*	8 (53)*	2 (13)*	3 (27)*	4 (36)*	0.16^†^

Results expressed as n (%). * Fisher’s test; ^†^ repeated-measures of analysis of variance test.

At the end of 12 weeks of treatment, children were classified according to the ICCS recommendation for therapeutic success. Only those showing no enuresis were considered as complete success of treatment, and the results are showed in table 2. There was no significant difference among the groups as to number of children presenting complete success.

**Table 2:** Number of children presenting complete success after treatment

	Group I	Group II	Group III	p value
Complete success	7 (58)*	11 (73)*	6 (55)*	0.59^†^

Results expressed as n (%). * Fisher’s test; ^†^ repeated-measures of analysis of variance test.

Two years after the end of the treatment, the results of questions made over the telephone are summarized in table 3.

**Table 3:** Number of children presenting complete success in treatment after 2-year follow-up

	Group I	Group II	Group III	p value
Complete success	5 (63)^*^	6 (75)^*^	3 (60)^*^	0.59^†^

Results expressed as n (%). * Fisher’s test; ^†^ repeated-measures of analysis of variance test.

The three groups showed no significant difference in the initial phase. After 12 weeks of treatment, there was a significant difference in relation to the treatment time (0.01), and not in relation to the groups.

## Discussion

The results showed that standard urotherapy alone or in combination with PFMT alone, or also in combination with oxybutynin, is effective in treatment of NMNE. The only study found in literature investigating the effect of standard urotherapy in children with monosymptomatic enuresis and NMNE was conducted by Mulders et al.^(7)^ In this retrospective study, 38 out of 98 children became completely dry during the day after 15 weeks of treatment; however, no data was reported about the effect of treatment on nocturnal enuresis episodes of these patients. This study corroborates the findings of improvement LUTS in response to this treatment.

Pelvic floor muscle training is a specific urotherapy intervention, and is known to improve LUTS symptoms in adults.^(8)^ It is also known that pelvic floor muscle work in all phases of micturition, avoiding leakage, suppressing a desire to void, and relaxing while the bladder is emptying.^(9)^ When LUTS are present, rehabilitation provides a motor control relearning, and improves their synergy function with the bladder. Although the benefits of PFM rehabilitation in adults have been well described, little is known about this modality of treatment for children with LUTS. Only one retrospective study in the evaluation of the effect of PFMT in NMNE was found, showing encouraging results with 64% of patients presenting improvement.^(5)^ To our knowledge, this study was the first to investigate the effect of PFMT prospectively, and compare it with an adequate control group. The results of this study showed that PFMT had no additional effect to standard urotherapy, probably because this modality also has a positive impact on the pelvic floor muscle function,^(7)^relaxing them while leading to a proper bladder empting. Furthermore, reduced caffeine intake is known to improve LUTS symptoms.^(10)^

Antimuscarinic drugs comprise a class that effectively and safely treats children with LUTS.^(11)^ They can improve NMNE symptoms by suppressing detrusor overactivity, which can be present in LUTS associated with NMNE. Moreover, antimuscarinic drugs alone can provide 54% of success rate.^(12)^ Hence, it is expected that the combination of this treatment modality with another, which is also known to be effective, such as urotherapy and/or PFMT, would lead to better results than single treatments. In this study, the group with standard urotherapy associated with PFMT and oxybutynin had better results in the reduction of enuresis, but they were not significant.

This study is limited to assessment tools. If an invasive evaluation were used, such as an urodynamic study, more variables would be collected and differences among the treatments would be detected. In addition, the 48-hour bladder diary was initially used as an inclusion criterion, and assessed again only 2 years after the end of treatment. If assessed right after the end of treatment, it would probably give relevant information. Even so, the results of this follow-up assessment will help future research, by showing that, even with the end of intervention, the changes provided by treatment continued to improve LUTS of these children.

The three treatment modalities used in this study showed encouraging results when used alone; thus, it is believed that the combination of these therapies would provide greater benefits in the treatment of NMNE. However, all modalities alone presented an average success rate of 50% in previous studies,^(5,7,12)^ and the sample size must be able to be precise and detect differences among the groups.

## Conclusion

All treatment modalities were effective in the treatment of nonmonosymptomatic nocturnal enuresis, and this corroborates the previous studies, but a larger sample size is needed to be able to detect differences among groups treated with combined treatments. Only duration of treatment was significant for improvement of non-monosymptomatic enuresis.
